# Seasonal distribution of *Anopheles funestus *chromosomal forms from Burkina Faso

**DOI:** 10.1186/1475-2875-8-239

**Published:** 2009-10-26

**Authors:** Wamdaogo M Guelbeogo, N'Fale Sagnon, Olga Grushko, Malgaouende A Yameogo, Daniela Boccolini, Nora J Besansky, Carlo Costantini

**Affiliations:** 1 Centre National de Recherche et de Formation sur le Paludisme, Ouagadougou, Burkina Faso; 2 Eck Institute for Global Health, Department of Biological Sciences, University of Notre Dame, Notre Dame, IN 46556 USA; 3 Department of Infectious, Parasitic, and Immunomediated Diseases, Istituto Superiore di Sanità, Rome, Italy; 4 Institut de Recherche pour le Développement, Research Unit UR016, Montpellier, France; 5 Current address: Organisation de Coordination pour la lutte contre les Endemies en Afrique Centrale, Yaoundé, Cameroun

## Abstract

**Background:**

Previous studies of *Anopheles funestus *chromosomal inversion polymorphisms in Burkina Faso showed large departures from Hardy-Weinberg equilibrium and linkage disequilibrium among inversions located on different chromosomes, implying the existence of two taxonomic units ("chromosomal forms") with limited genetic flow. One chromosomal form, named Folonzo, is highly polymorphic for alternative rearrangements of 3R*a*, 3R*b*, 2R*a*, and 3L*a*; the other, Kiribina, is predominantly characterized by the standard arrangement of these inversions. To investigate the temporal distribution of these chromosomal forms, further collections were carried out in two villages near Ouagadougou where they are found in sympatry.

**Methods:**

Chromosomal karyotypes were determined from indoor-resting, half-gravid females sampled within and across six breeding seasons, from December 1998 to April 2007.

**Results:**

As expected, the pattern of chromosomal polymorphism in *An. funestus *was consistent with assortatively mating Folonzo and Kiribina forms. When samples were assigned to each chromosomal form, their relative abundance varied within successive breeding seasons in a repeating pattern of temporal variability. Relative abundance of the Folonzo form was correlated with climatic variables related to temperature and rainfall.

**Conclusion:**

The relative abundance of Folonzo and Kiribina forms of *An. funestus *likely reflects different larval ecologies that are linked to varying climatic conditions. Further analysis of the bionomics of these vectors is recommended in light of its relevance to vector control.

## Background

Malaria remains a major health concern in Africa today. This situation is due to the presence of three efficient vectors in subgenus *Cellia--Anopheles gambiae*, its sibling species *Anopheles arabiensis *and *Anopheles funestus*. These species co-occur geographically across most of sub-Saharan Africa and can inhabit the same villages, shelter in the same houses, and blood-feed on the same individuals. Vector control efforts are complicated not only by the spread of resistance to insecticides, but also by behavioural and genetic variation within and between species. Among the challenges of efficient vector control are cryptic barriers to gene flow among populations of the same species that can arise as a consequence of differential adaptations to heterogeneities in the environment. This challenge applies not only to the introduction and spread of "refractoriness genes" in wild mosquito populations, but also to the use of bed nets whose efficacy could be lessened by exophilic and exophagic vector sub-populations [1]. Sustained vector control efforts across Africa will benefit from knowledge of the extent of genetic diversity in natural populations, how it is distributed in time and space, and how it is generated and maintained among vector populations [2-4].

*An. funestus *abounds during the dry season and is less rain-dependant than *An. gambiae s.l.*, owing to the tendency to breed in permanent or semi-permanent swamps or pools [5]. As a result, it is considered as a vector that bridges malaria transmission across the dry season. However, it has long been noted that *An. funestus *is a highly polymorphic species whose populations could be structured. Evidence of genetic heterogeneities within this mosquito were revealed during early cytogenetic investigations [6]. Polymorphic inversions are found in several populations in East [7], South [6,8], Central [3,9], and West Africa [10-12]. Some of the inversion systems are specific to each locality. Echoing the discovery of complexities in other isomorphic anopheline species, cytogenetic analysis of *An. funestus *in Burkina Faso revealed large departures from Hardy-Weinberg equilibrium and linkage disequilibrium among inversions located on different chromosomes. These results led to the recognition of two chromosomal forms, namely "Folonzo" and "Kiribina," characterized by contrasting degrees of chromosomal polymorphism and limited amounts of gene flow, considered indicative of an incipient speciation process [10]. This hypothesis was reinforced in a detailed cytogenetic analysis during three consecutive seasons in a pair of villages less than 2 km apart near Ouagadougou [13]. More recently, further evidence for genetic differentiation between the two chromosomal forms in Burkina Faso was found using molecular markers: 16 microsatellite loci dispersed over the chromosome complement, and the ND5 region of mtDNA [14]. The geographic extent of this diversification in *An. funestus *is uncertain, as studies of the two chromosomal forms in Cameroon and Senegal detected no evidence of genetic differentiation beyond that accounted for by geographical isolation [3,15].

The spatio-temporal pattern of these diverging entities and the underlying factors governing the pattern remain unclear. Ultimately, assessment of both spatial and temporal components of the genetic structure of a species is necessary to identify factors that influence the genetic variability and relationships among its populations. Monitoring populations over time, the objective of the present study, allows determination of the degree of stability of spatial structuring within a population or local geographical area. Toward this end, a temporal analysis of *An. funestus *chromosomal forms was performed during seven breeding seasons in villages where the two forms are sympatric.

## Methods

### Study area

Mosquito collections were carried out in two rural villages near Ouagadougou, Burkina Faso: Koubri (12°11'54"N; 1°23'43"W) and Kuiti (12°11'36"N; 1°23'11"W). The two villages lie in the West African sudan savannah vegetation belt, and are located ~2 km apart on opposite margins of a swamp [for detailed map, see [13]]. The area receives 750 mm of rain per year, mainly falling during the rainy season from July until September. The breeding season of *An. funestus *starts at the end of the rainy season (September), extends throughout the cool dry season (October-February), and ends in April, mid-way through the hot dry season (March-May).

### Mosquito collection

**Figure 1:**
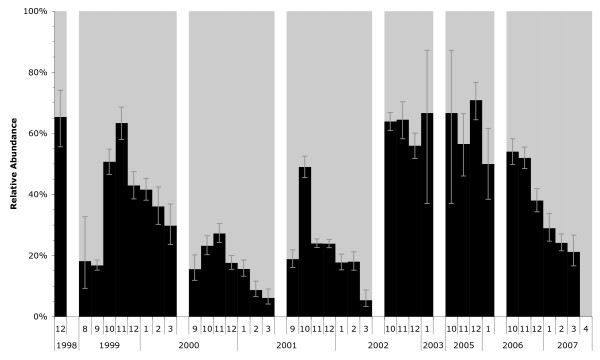
**Relative frequencies (± standard errors) of *An. funestus *Folonzo and Kiribina chromosomal forms by year and month of collection**. Black bars and gray bars refer to Folonzo and Kiribina, respectively.

For detailed analysis of chromosomal forms within breeding seasons, indoor resting collections were made in three consecutive years from August 1999 to March 2002 (see Figure 1). Mosquitoes were collected three times per week manually or by spray-catch. Further details of these collections are presented in references [13,14]. Based on preliminary karyotype analysis of these collections, further collections were performed during three additional years, at the anticipated peak period of chromosomal polymorphism beginning in October and continuing through January or beyond.

### Specimen processing

Adult *An. funestus *group mosquitoes were sorted morphologically in the field [5,16]. Half-gravid females were immediately dissected; ovaries were preserved individually in 1.5 ml microcentrifuge tubes using Carnoy's fixative and the carcass was placed in a correspondingly labelled tube with desiccant. After fixation, polytene chromosomes were squashed [17]. The slides were examined under a phase-contrast microscope, and chromosomal arrangements were scored with reference to the cytogenetic map [18]. Assignment to chromosomal form was based on the algorithm of Costantini *et al *[10] modified by Guelbeogo *et al *[13].

### Weather data

Climatic variables (monthly temperature, relative humidity, and rainfall) were provided by the National Meteorological Agency through their meteorological station located in the village of Koubri.

### Data analysis

As previous studies showed genetic homogeneity within chromosomal forms collected from Koubri and Kuiti [13,14], analysis was performed on pooled samples from these two adjacent villages. The Spearman Rank correlation coefficient was used to test the association between the relative abundance (i.e. relative frequency--hereafter used synonymously) of Folonzo and several climatic variables: total annual and monthly rainfall, monthly relative humidity (maximum and minimum), and monthly temperature (minimum, maximum and mean). Correlation was tested between the pooled relative abundance of the *An. funestus *Folonzo form in Oct-Dec of each year with respect to the corresponding climatic variables during the preceding (Jan-Sep) or concurrent (Oct-Dec) months. Where a significant correlation was found, the relationship between those variables and Folonzo relative abundance was parameterized by logistic regression. Analyses were run in R v. 2.9.0 [19].

### Ethical consideration

The study protocol was reviewed and approved by the institutional health ethical review board of Burkina Faso (code N° 2007-034). Participants were explained the study procedures, benefits and risks and their consent was obtained before collecting mosquito in their settlements.

## Results

A total of 6,582 indoor-resting *An. funestus *adults were successfully scored chromosomally. Of these, 1,959 and 4,623 were assigned to Folonzo and Kiribina forms, respectively.

In the three consecutive breeding seasons for which monthly samples were taken, relative frequencies of the chromosomal forms cycled in a repeatable pattern (Figure 1). The Folonzo form achieved its greatest relative frequencies in October-December. Although other seasonal collections were less comprehensive, the chromosomal data were consistent with this same pattern of Folonzo peaking in relative abundance following the rains.

**Figure 2:**
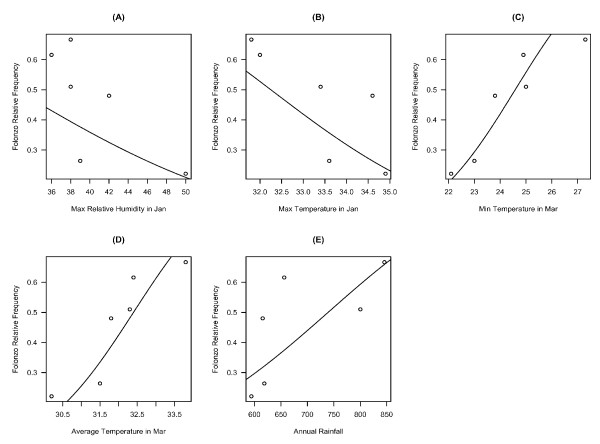
**Relationship between five climatic variables and *An. funestus *Folonzo relative frequency (plots A-E), as described by logistic regression lines**.

Beyond this general trend, the average relative frequency of the two chromosomal forms varied between breeding seasons. These temporal patterns of abundance suggest the influence of climatic variables. The possible relationship between Folonzo relative frequency during the peak months of abundance (Oct-Dec) and rainfall, relative humidity and temperature was explored with reference to months preceding the breeding season in question. A significant correlation was found with four monthly variables (Table 1). During January, maximum relative humidity and temperature were negatively correlated with Folonzo relative abundance in the following breeding season. Similarly in March, mean and minimum temperatures were positively correlated with Folonzo relative frequency afterward. Overall, annual rainfall amount was positively correlated with Folonzo relative abundance. Figure 2 shows the relationships between these five variables and the Folonzo form frequency as described by logistic regression lines. The corresponding equations are provided in Table 2. It is acknowledged that some of these significant associations might be spurious because the climatic variables used are not independent, and a large number of tests were performed. However, in adopting the approach recommended by Moran [20] for ecological research, a correction for multiple statistical tests was not applied at this exploratory stage. The fact that five tests gave fairly low probability values suggests that a general pattern of association between climate and Folonzo relative abundance is likely to be valid, but further studies will be required to evaluate the relative importance of these and other variables to the observed pattern.

**Table 1:** Correlation between the pooled relative abundance of the *An. funestus *Folonzo form in Oct-Dec of each year and the corresponding climatic variables during the preceding (Jan-Sep) or concurrent (Oct-Dec) months (coefficients, top tier; P-values, bottom tier).

**Variable**	**Jan**	**Feb**	**Mar**	**Apr**	**May**	**Jun**	**Jul**	**Aug**	**Sep**	**Oct**	**Nov**	**Dec**	**Total**
**Rainfall**	NA	NA	0.563	0.594	-0.605	0.049	0.668	0.615	0.738	-0.248	NA	NA	**0.854**
**Rainy Days**	NA	NA	0.621	0.730	-0.335	-0.475	-0.385	0.621	0.236	-0.355	NA	NA	0.072
**Max RH**	**-0.772**	0.265	0.393	0.377	-0.548	-0.361	-0.359	0.213	0.151	0.109	0.274	0.550	
**Min RH**	-0.217	0.164	0.439	-0.182	-0.512	-0.082	0.235	0.596	0.661	0.210	-0.749	-0.305	
**Mean Temp**	-0.503	0.695	**0.945**	-0.019	0.254	0.433	0.427	-0.256	0.093	-0.464	-0.551	0.226	
**Min Temp**	-0.174	0.711	**0.931**	0.025	0.177	0.531	0.667	0.135	0.294	-0.752	-0.348	0.276	
**Max Temp**	**-0.910**	0.697	0.600	-0.629	0.000	0.160	0.355	-0.569	0.029	-0.332	-0.428	0.162	
													
**Rainfall**	NA	NA	0.268	0.297	0.242	1.000	0.136	0.175	0.058	0.658	NA	NA	**0.033**
**Rainy Days**	NA	NA	0.188	0.103	0.564	0.354	0.497	0.173	0.803	0.461	NA	NA	0.919
**Max RH**	**0.036**	0.612	0.552	0.461	0.257	0.497	0.485	0.681	0.868	0.827	0.538	0.321	
**Min RH**	0.742	0.870	0.321	0.774	0.295	0.868	0.827	0.249	0.123	0.658	0.059	0.518	
**Mean Temp**	0.354	0.103	**0.003**	0.913	0.714	0.354	0.497	0.784	1.000	0.354	0.257	0.803	
**Min Temp**	0.497	0.103	**0.017**	0.919	0.700	0.419	0.242	0.784	0.538	0.103	0.499	0.695	
**Max Temp**	**0.017**	0.136	0.257	0.136	0.919	0.827	0.497	0.257	1.000	0.564	0.461	0.803	

NA, cannot be calculated because all relevant values were zero.

Significant values shown in bold.

**Table 2:** Parameters (± standard errors) of the regression lines shown in Figure 2 (see text for details).

**Variable**	**Intercept**	**Slope**
Max RH in Jan	2.424	-0.075
	(± 0.337)	(± 0.008)
Max Temp in Jan	14.100	-0.437
	(± 1.295)	(± 0.039)
Min Temp in Mar	-13.664	0.556
	(± 0.772)	(± 0.033)
Mean Temp in Mar	-24.747	0.764
	(± 1.632)	(± 0.052)
Annual Rainfall	-4.579	0.006
	(± 0.343)	(± 0.001)

NA, cannot be calculated because all relevant values were zero.

## Discussion

The three main components of the malaria vectorial system in tropical Africa have been observed to cycle in relative abundance according to seasonal ecological conditions [5], in a pattern often described as a relay due to the temporal succession of *An. gambiae *followed by *An. arabiensis *and finally *An. funestus*. For the first two mosquito vectors, the positive relationship between rainfall and abundance is unsurprising, because these species typically breed in temporary rain-dependent pools and puddles. Because *An. funestus *characteristically breeds in large permanent or semi-permanent pools (*e.g*., swamps and irrigated rice fields), rain-dependent fluctuations in its abundance may seem less obvious. However, emergent vegetation--particularly around the margins of breeding sites--is considered one of the more important ecological features for this species [5]. Especially in the dry savannah where rainfall is restricted to one season, both the physical extent of the site as well as the growth of vegetation depends upon rainfall and the level of the water table. Accordingly, potential *An. funestus *larval breeding sites are not uniformly distributed during the year.

Available breeding sites for *An. funestus *in the immediate study area of Kuiti and Koubri include both a permanent swamp and rice fields. There is considerable modification of the swamp at the end of rainy season because decreasing water levels lead to the appearance and disappearance of different types of breeding sites. In addition, human activities surrounding rice cultivation--the flooding of the fields in June/July and their harvest in October/November--destroy and create new breeding sites that may favour one or the other of the alternative chromosomal forms. Because the mosquitoes analyzed during this study were collected as adults, there is no direct evidence linking the increased relative abundance of the Folonzo form to features of these breeding sites. However, by analogy to habitat segregation in the *An. gambiae *complex [21-24], it is possible to hypothesize that niche partitioning exists between the chromosomal variants of *An. funestus*. Consistent with this idea, Costantini *et al *[10] reported high frequencies of Folonzo in villages associated with water reservoirs containing natural emerging and floating vegetation, whereas high frequencies of Kiribina were documented in villages with large scale rice crop areas. This suggests that Kiribina prefers breeding in rice fields or out-competes Folonzo in these artificial, anthropogenic habitats; the converse may be true of Folonzo in sites containing natural vegetation, such as swamps. Testing of this hypothesis crucially depends upon the discovery and application of molecular markers that can distinguish Folonzo and Kiribina forms, for two reasons. From a practical standpoint, only the polytene chromosomes extracted from half-gravid adult females can be used in the recognition of the *An. funestus *chromosomal forms presently [13], posing a serious impediment if not roadblock to research on their larval ecology. Of potential theoretical importance is the correspondence between chromosomally recognized variants and true assortatively mating and biologically relevant reproductive units. As is apparent in the case of *An. gambiae*, such correspondence is far from perfect, reflecting the fact that different reproductive units share chromosomal variants to some extent [23-26]. If a similar situation exists within *An. funestus *as hypothesized, the discovery of molecular markers better reflecting reproductive discontinuities will be an important prerequisite to further research defining the ecological mechanisms driving diversification in this lineage. It is hoped that the recently approved whole genome sequencing of several *Anopheles *species including *An. funestus *[27] will offer the requisite tools to help overcome this problem.

Coluzzi [2,28] has emphasized the significance of such a genetically heterogeneous malaria vectorial system to malaria epidemiology and control in Africa, because it results in very high and stable disease transmission. The operational value of distinguishing between isomorphic vector and non-vector species can be taken for granted, but Coluzzi [2,28] also stressed the less obvious relevance of distinctions between reproductively isolated siblings or incipient species presumed to share high vectorial potential. The core of his argument is that the stable co-existence of two taxa implies some form of biological divergence, the absence of which would lead to costly and ultimately unsustainable competition. Biological divergence could be reflected in such epidemiologically relevant factors as vectorial capacity and response to vector control strategies. In fact, it is conceivable that the Folonzo and Kiribina forms do differ in their behavioural responses to environmental heterogeneities. In a remarkable parallel with *An. gambiae *[25,29,30], inversion frequencies in *An. funestus *populations from Cameroon vary clinally from mesic forest regions in the south where inverted arrangements are fixed to xeric regions in the north where the standard karyotype is fixed [3]. These findings are in agreement with the positive association between Folonzo relative frequency and total rainfall that was found in the present study. This suggests that Folonzo would be better adapted to environments with a higher degree of water vapour saturation and Kiribina would be better adapted to periods with a higher saturation deficit, which could impact epidemiologically important behaviours such as indoor resting and biting. This pattern of chromosomal variation is also consistent with the present findings in an area of sympatry, where the Folonzo form breeds preferentially during the months following the end of the rainy season. Answering Coluzzi's [2] call for vector analysis in the case of *An. funestus *thus will require a wide range of genotypic and phenotypic information preferably based on parallel adult and larvae sampling.

## Competing interests

The authors declare that they have no competing interests.

## Authors' contributions

CC, NS and NB conceived the study. WG performed the field collection. NS provided logistical support throughout the study. WG, OG, DB and MAY performed the karyotyping. WG and CC analysed results. WG, NB and CC wrote the manuscript. All authors read and approved the final manuscript.
